# Marital status and survival in epithelial ovarian cancer patients: a SEER-based study

**DOI:** 10.18632/oncotarget.21648

**Published:** 2017-10-06

**Authors:** Xinyu Wang, Xi Li, Shaofei Su, Meina Liu

**Affiliations:** ^1^ Department of Biostatistics, Public Health College, Harbin Medical University, Harbin, China

**Keywords:** epithelial ovarian cancer, marital status, cancer survival, SEER

## Abstract

Marital status has been proved to be correlated to the survival of patients in various cancer types, except for that in the large female population of epithelial ovarian cancer (EOC). In this study, we retrospectively extracted 10905 eligible EOC patients from the Surveillance, Epidemiology, and End Results (SEER) database in the period from 2004 to 2012. We categorized marital status as married, divorced/separated, widowed, and never married. Chi-square test was used to investigate the association between marital status and other variables. The Kaplan-Meier test was adopted to compare survival curves of different groups. Multivariate Cox regression analyses were conducted to estimate the effect of marital status on overall survival (OS) and epithelial ovarian cancer-specific survival (EOCSS). To explore how marital status affected patients diagnosed at the same stage, we further performed subgroup analyses according to TNM stage. The results showed that marital status was an independent predictor for OS and EOCSS. Subgroup analyses indicated that the relationship between marital status and prognosis varied according to different conditions. Widowed patients had poorer prognosis than the other groups in most conditions, while the never married group showed similar risk of mortality as the married ones.

## INTRODUCTION

Epithelial ovarian cancer (EOC) is a clinically and biologically heterogeneous class of tumors including several major subtypes: serous, mucinous, endometrioid, and clear cell [1]. Although advances in diagnosis and treatment have been achieved in recent decades, age-standardized 5-year survival rate from ovarian cancer for all histological groups combined was around 30–40% in most countries from 1995 to 2009 [2]. It has become the fifth leading cause of cancer death in women in the U.S. In 2017, it is estimated that 22,440 new cases of ovarian cancer will be diagnosed and 14,080 deaths due to ovarian cancer will occur [3].

Previous studies have identified that women with a family history of ovarian cancer are at an increased risk of ovarian cancer [4]. Moreover, certain factors like the type, grade, and stage of the cancer, patients’ age and general health all affect the prognosis of EOC patients. Micro-level studies have also helped to better and more precisely predict the survival of ovarian cancer [5]. Among the studies, however, marital status, which plays an important role in women’s physical and mental health, has rarely been investigated for its effect on the EOC prognosis. In recent years, many researches have demonstrated that marital status independently predicts the survival of gastric cancer [6–8], colorectal cancer [9, 10], liver cancer [11], pancreatic cancer [12], and several other types of cancer [13–16]. Identifying the relationship between the marital status and the survival of EOC would help researchers, doctors, as well as policy makers better cope with the increasing trend of mortality rate.

Therefore, we performed a comprehensive population-based analysis to clarify the prognostic significance of marital status on the survival of EOC patients. We used data in 2004-2012 from the US Surveillance, Epidemiology and End Results (SEER) cancer registry program to investigate the association of overall survival and ovarian cancer-specific survival with marital status and further analyzed the association stratified by stage.

## RESULTS

### Patient baseline characteristics

A total of 10905 eligible EOC patients were identified during the study period (from 2004 to 2012) in the SEER database. Among them, 5919 (54.28%) were married, 1268 (11.63%) were divorced or separated, 1733 (15.89%) were widowed, and 1985 (18.20%) were never married. Table 1 represents the summary of the subgroups of each variable and the relationship between each variable and marital status. Significant differences were observed in most of the comparisons. Specifically, widowed patients were more likely to be over 80 years (41.32%), while most of the never married patients were less than 60 years (67.41%). White patients accounted for the majority of each marital group, but the proportion of black patients was slightly higher in never married group (13.50%) than that in other groups. The distribution of marital status in diagnosis period of 2004-2008 and 2009-2012 was not statistically different (*P*=0.0612). Widowed patients were more likely to be diagnosed at stage IV (40.74%), while never married patients had the highest proportion among the four groups of being diagnosed at stage I. Moreover, widowed patients had the lowest proportion of receiving any type of surgery (63.24%), while the married had the highest (90.13%), compared with other groups.

**Table 1:** Baseline characteristics of EOC patients (n, %)

Characteristic	Total	Married	Divorced/Separated	Widowed	Never married	P value
Marital status	10905(100.00)	5919(54.28)	1268(11.63)	1733(15.89)	1985(18.20)	
Age						<0.0001
<60	5065(46.45)	2994(50.58)	595(46.92)	138(7.96)	1338(67.41)	
60-69	2707(24.82)	1648(27.84)	371(29.26)	311(17.95)	377(18.99)	
70-79	1882(17.26)	925(15.63)	225(17.74)	568(32.78)	164(8.26)	
≥80	1251(11.47)	352(5.95)	77(6.07)	716(41.32)	106(5.34)	
Race						<0.0001
White	8956(82.13)	5021(84.83)	1024(80.76)	1404(81.02)	1507(75.92)	
Black	846(7.76)	267(4.51)	144(11.36)	167(9.64)	268(13.50)	
Others	1103(10.11)	631(10.66)	100(7.89)	162(9.35)	210(10.58)	
Diagnosis year						0.0612
2004-2008	5709(52.35)	3076(51.97)	670(52.84)	954(55.05)	1009(50.83)	
2009-2012	5196(47.65)	2843(48.03)	598(47.16)	779(44.95)	976(49.17)	
Grade						<0.0001
I	732(6.71)	415(7.01)	57(4.50)	58(3.35)	202(10.18)	
II	1365(12.52)	776(13.11)	148(11.67)	145(8.37)	296(14.91)	
III	3751(34.40)	2133(36.04)	468(36.91)	516(29.77)	634(31.94)	
IV	1966(18.03)	1148(19.40)	240(18.93)	252(14.54)	326(16.42)	
Unknown	3091(28.34)	1447(24.45)	355(28.00)	762(43.97)	527(26.55)	
TNM stage						<0.0001
I	2512(23.04)	1421(24.01)	249(19.64)	244(14.08)	598(30.13)	
II	992(9.10)	565(9.55)	106(8.36)	137(7.91)	184(9.27)	
III	4290(39.34)	2452(41.43)	503(39.67)	646(37.28)	689(34.71)	
IV	3111(28.53)	1481(25.02)	410(32.33)	706(40.74)	514(25.89)	
Histological type						<0.0001
Serous	5544(50.84)	3182(53.76)	702(55.36)	803(46.34)	857(43.17)	
Mucinous	604(5.54)	310(5.24)	73(5.76)	64(3.69)	157(7.91)	
Clear-cell	780(7.15)	446(7.54)	75(5.91)	62(3.58)	197(9.92)	
Endometrioid	1162(10.66)	657(11.10)	116(9.15)	109(6.29)	280(14.11)	
Others	2815(25.81)	1324(22.37)	302(23.82)	695(40.10)	494(24.89)	
Surgery						<0.0001
No	1707(15.65)	584(9.87)	206(16.25)	637(36.76)	280(14.11)	
Yes	9198(84.35)	5335(90.13)	1062(83.75)	1096(63.24)	1705(85.89)	

### Effect of marital status on overall and cancer-specific survival

**Figure 1:**
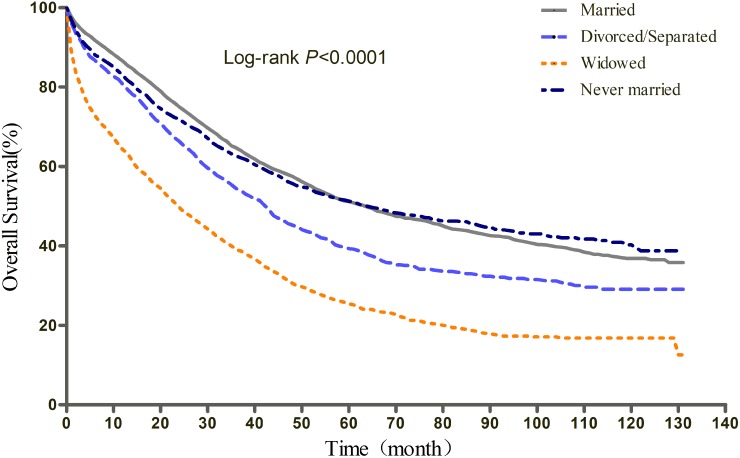
Kaplan-Meier curves of the effect of marital status on overall survival (OS)

**Figure 2:**
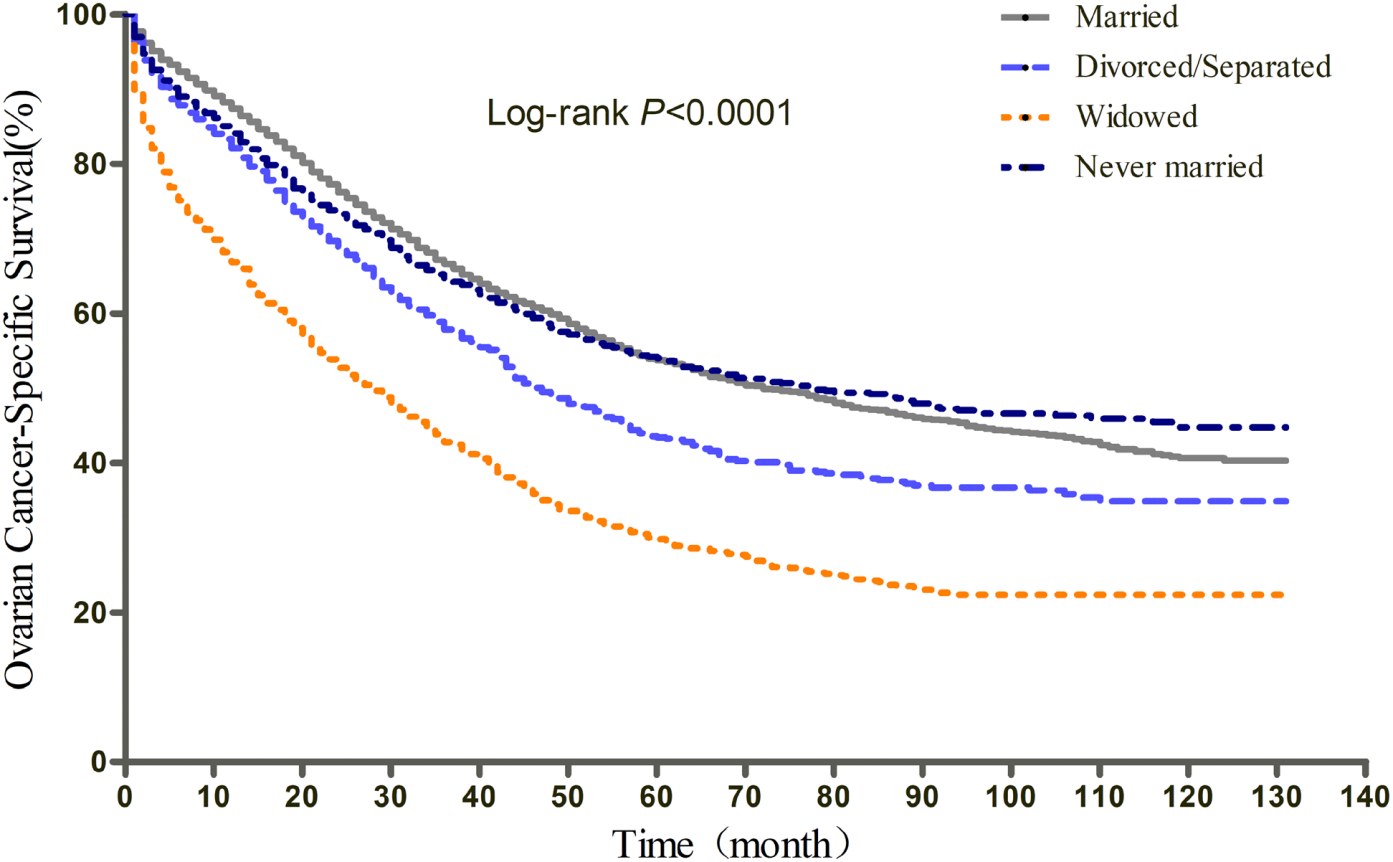
Kaplan-Meier curves of the effect of marital status on epithelial ovarian cancer-specific survival (EOCSS)

The results of Kaplan-Meier tests and multivariate Cox analyses of the effect of marital status on OS and EOCSS were shown in Table 2 and Table 3, respectively. The 5-year OS rate was 51.11% for the married, 39.30% for the divorced/separated, 51.28% for the never married, and 25.40% for the widowed (log-rank test *P*<0.0001) (Figure 1). After adjusting for other confounding factors with multivariate Cox regression, marital status was found to be an independent predictor of OS. Divorced/separated (HR=1.21, 95%CI: 1.11, 1.31), widowed (HR=1.12, 95%CI: 1.04, 1.20), and never married (HR=1.11, 95%CI: 1.03, 1.19) patients had an increased risk of mortality compared with married patients. In terms of EOCSS, the 5-year EOCSS rate was 53.72% for married patients, 43.42% for divorced/separated patients, 29.81% for widowed, and 54.02% for never married patients (log-rank test *P*<0.0001) (Figure 2). Similarly, after adjusting for all covariates, marital status was still identified as significantly associated with the EOCSS. Divorced/separated (HR=1.15, 95%CI: 1.06, 1.26), widowed (HR=1.11, 95%CI: 1.03, 1.20), and never married (HR=1.10, 95%CI: 1.02, 1.19) patients had an increased risk of EOC-specific mortality compared with married patients.

**Table 2:** Univariate and multivariate analyses of overall survival (OS)

Variables	5-year OS (%)	Univariate analysis		Multivariate analysis	
		Log-rank	*P*	HR(95%CI)	*P*
Marital status		606.08	<0.0001		
Married	51.11			Ref.	
Divorced/separated	39.30			1.21(1.11, 1.31)	<0.0001
Widowed	25.40			1.12(1.04, 1.20)	0.0040
Never married	51.28			1.11(1.03, 1.19)	0.0070
Age		1851.17	<0.0001		
<60	59.23			Ref.	
60-69	44.94			1.19(1.11, 1.27)	<0.0001
70-79	31.40			1.48(1.38, 1.60)	<0.0001
≥80	14.29			2.11(1.93, 2.31)	<0.0001
Race		98.70	<0.0001		
White	45.83			Ref.	
Black	33.34			1.20(1.10, 1.31)	<0.0001
Others	53.51			0.97(0.88, 1.06)	0.4693
Diagnosis year		6.68	0.0098		
2004-2008	44.86			Ref.	
2009-2012	45.57			0.94(0.89, 0.99)	0.0180
Grade		1121.83	<0.0001		
I	84.75			Ref.	
II	67.33			1.47(1.20, 1.80)	0.0002
III	43.17			1.78(1.47, 2.15)	<0.0001
IV	44.92			1.63(1.34, 1.99)	<0.0001
Unknown	30.12			1.65(1.36, 2.01)	<0.0001
TNM stage		3099.93	<0.0001		
I	86.11			Ref.	
II	67.00			2.56(2.20, 2.97)	<0.0001
III	37.26			5.45(4.83, 6.14)	<0.0001
IV	18.26			7.25(6.41, 8.21)	<0.0001
Histological type		1140.85	<0.0001		
Serous	40.09			Ref.	
Mucinous	68.05			1.71(1.46, 1.99)	<0.0001
Clear-cell	64.33			1.39(1.22, 1.58)	<0.0001
Endometrioid	81.94			0.66(0.57, 0.76)	<0.0001
Others	31.65			1.09(1.02, 1.16)	0.0105
Surgery		4068.36	<0.0001		
No	7.03			Ref.	
Yes	52.81			0.38(0.35, 0.41)	<0.0001

**Table 3:** Univariate and multivariate analyses of epithelial ovarian cancer-specific survival (EOCSS)

Variables	5-year CSS (%)	Univariate analysis		Multivariate analysis	
		Log-rank	*P*	HR(95%CI)	*P*
Marital status		483.44	<0.0001		
Married	53.72			Ref.	
Divorced/separated	43.42			1.15(1.06, 1.26)	0.0009
Widowed	29.81			1.11(1.03, 1.20)	0.0087
Never married	54.02			1.10(1.02, 1.19)	0.0163
Age		1431.58	<0.0001		
<60	61.17			Ref.	
60-69	47.70			1.13(1.06, 1.22)	0.0005
70-79	35.83			1.35(1.24, 1.46)	<0.0001
≥80	18.85			1.82(1.65, 2.00)	<0.0001
Race		84.07	<0.0001		
White	48.88			Ref.	
Black	36.98			1.14(1.04, 1.25)	0.0064
Others	57.63			0.93(0.84, 1.03)	0.1497
Diagnosis year		6.33	0.0119		
2004-2008	48.06			Ref.	
2009-2012	49.22			0.93(0.88, 0.99)	0.0165
Grade		1095.98	<0.0001		
I	88.01			Ref.	
II	71.57			1.53(1.22, 1.92)	0.0002
III	46.18			1.91(1.54, 2.37)	<0.0001
IV	47.42			1.76(1.41, 2.20)	<0.0001
Unknown	33.12			1.77(1.42, 2.21)	<0.0001
TNM stage		3104.52	<0.0001		
I	89.74			Ref.	
II	72.00			3.08(2.59, 3.65)	<0.0001
III	39.93			7.23(6.29, 8.31)	<0.0001
IV	20.35			9.67(8.38,11.15)	<0.0001
Histological type		1131.21	<0.0001		
Serous	42.97			Ref.	
Mucinous	71.14			1.78(1.51, 2.10)	<0.0001
Clear-cell	67.74			1.45(1.26, 1.66)	<0.0001
Endometrioid	86.11			0.58(0.50, 0.69)	<0.0001
Others	34.73			1.10(1.03, 1.17)	0.0068
Surgery		3847.60	<0.0001		
No	8.59			Ref.	
Yes	55.99			0.36(0.33, 0.39)	<0.0001

Besides, we found that the hazard ratio increased with the growth of age; black patients were associated with poorer OS and EOCSS; compared with the patients with the histological type of serous, patients with mucinous and clear-cell cancer had increased risk of overall and EOC-specific mortality, while patients with endometroid cancer had reduced risk of the both; the hazard ratio increased as the TNM stage at diagnosis advanced; as expected, patients who underwent surgery had better prognosis than that of the ones who did not.

### Subgroup analyses of patients stratified by TNM stage

**Figure 3:**
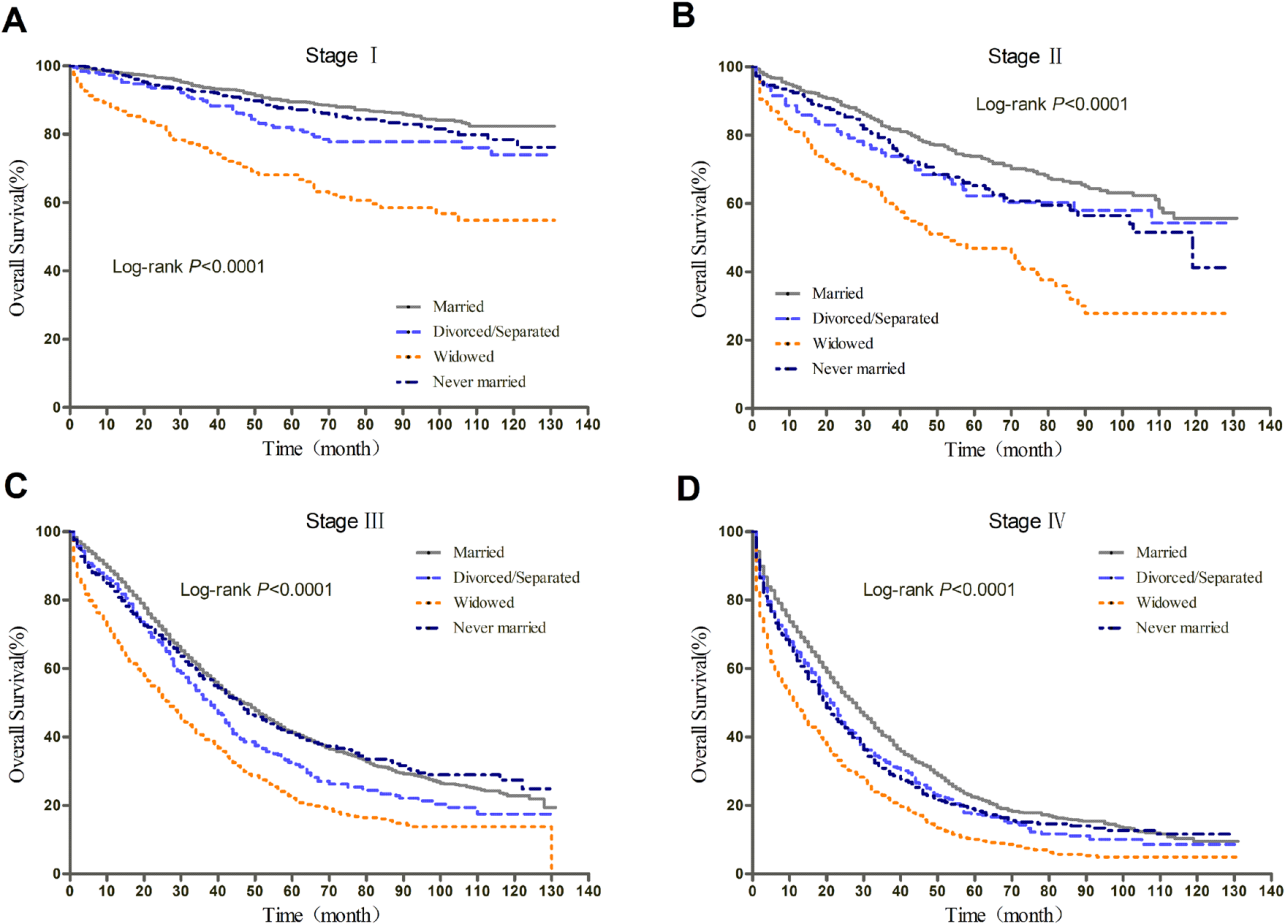
Kaplan-Meier curves of the effect of marital status on OS for all patients stratified by stage

**Figure 4:**
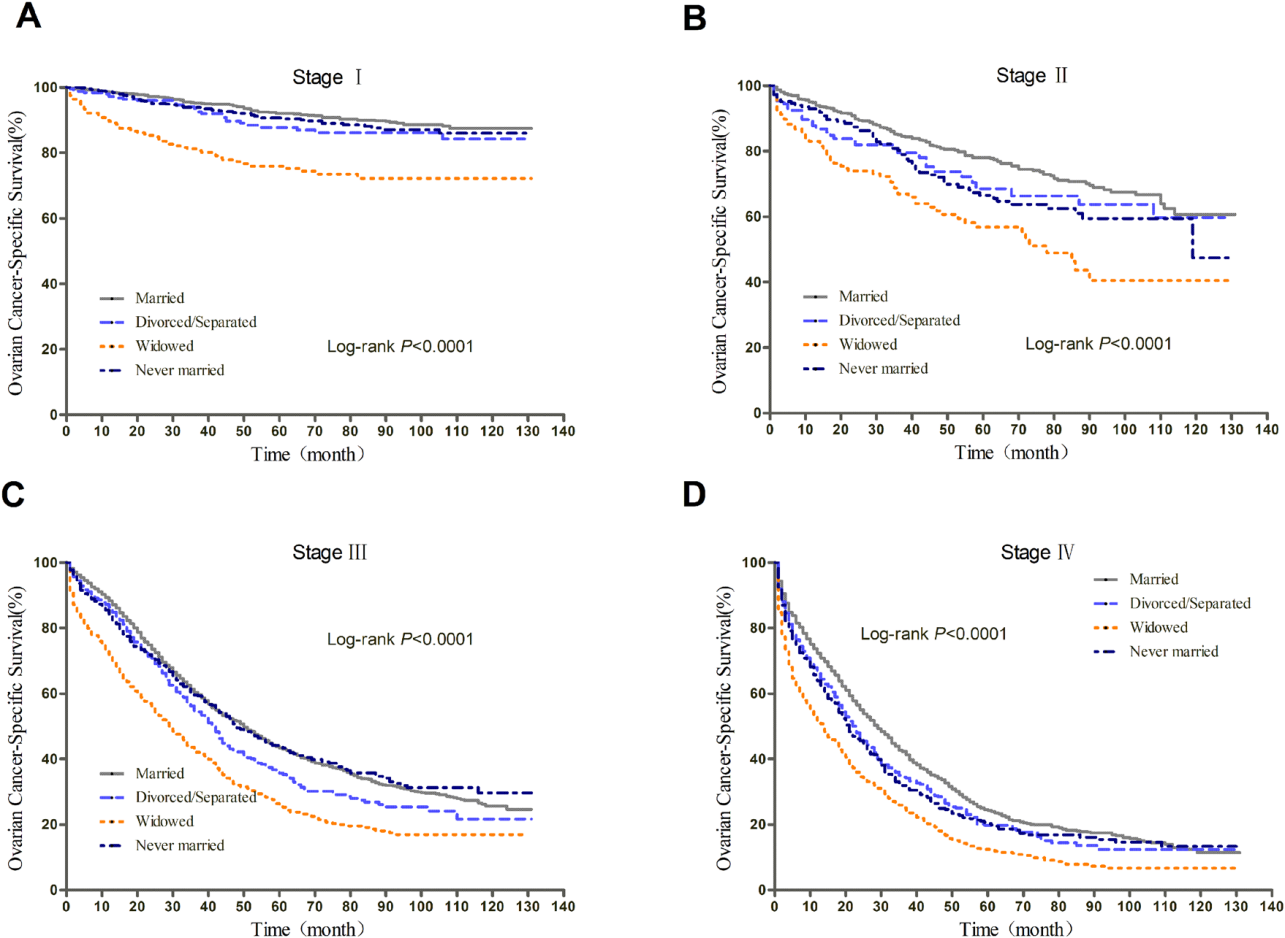
Kaplan-Meier curves of the effect of marital status on EOCSS for all patients stratified by stage

We further explored the effect of marital status on OS and EOCSS, stratified by TNM stage. The stage-specific survival curves of the OS and EOCSS of different marital status were shown in Figure 3 and Figure 4, respectively. The results of stage-stratified Cox regression were summarized in Table 4 for OS and Table 5 for EOCSS.

**Table 4:** Subgroup analyses stratified by TNM stage for all EOC patients (OS)

	Stage I	Stage II	Stage III	Stage IV
Marital status				
Married	Ref.	Ref.	Ref.	Ref.
Divorced/separated	1.56(1.12, 2.18) ^a^	1.07(0.75, 1.54)	1.21(1.08, 1.37)^a^	1.13(1.00, 1.28)
Widowed	1.52(1.11, 2.08)^a^	1.38(1.01, 1.87)^b^	1.13(1.01, 1.27)^b^	1.07(0.96, 1.20)
Never married	1.19(0.91, 1.57)	1.54(1.15, 2.06)^a^	1.04(0.93, 1.16)	1.11(0.99, 1.25)
Age				
<60	Ref.	Ref.	Ref.	Ref.
60-69	1.06(0.79, 1.43)	1.05(0.78, 1.43)	1.21(1.09, 1.33)^a^	1.12(1.00, 1.24)^b^
70-79	2.37(1.75, 3.22)^a^	2.10(1.53, 2.88)^a^	1.59(1.43, 1.78)^a^	1.19(1.06, 1.34)^a^
≥80	5.03(3.56, 7.10)^a^	3.54(2.51, 4.99)^a^	2.12(1.84, 2.43)^a^	1.62(1.41, 1.86)^a^
Race				
White	Ref.	Ref.	Ref.	Ref.
Black	1.26(0.88, 1.80)	1.43(0.97, 2.09)	1.20(1.04, 1.38)^b^	1.07(0.94, 1.22)
Others	0.78(0.56, 1.10)	0.84(0.56, 1.25)	0.99(0.86, 1.13)	1.03(0.89, 1.18)
Diagnosis year				
2004-2008	Ref.	Ref.	Ref.	Ref.
2009-2012	0.88(0.69, 1.11)	1.02(0.81, 1.30)	0.92(0.85, 1.00)	0.96(0.88, 1.04)
Grade				
I	Ref.	Ref.	Ref.	Ref.
II	1.46(1.00, 2.13)	1.17(0.66, 2.08)	1.51(1.08, 2.11)^b^	1.38(0.87, 2.17)
III	1.74(1.18, 2.55)^a^	1.60(0.92, 2.77)	1.90(1.39, 2.62)^a^	1.32(0.86, 2.02)
IV	1.56(0.98, 2.48)	1.22(0.67, 2.21)	1.76(1.28, 2.44)^a^	1.26(0.81, 1.95)
Unknown	1.66(1.12, 2.46)^b^	1.66(0.93, 2.97)	1.84(1.33, 2.55)^a^	1.21(0.79, 1.86)
Histological type				
Serous	Ref.	Ref.	Ref.	Ref.
Mucinous	0.94(0.66, 1.34)	1.05(0.60, 1.84)	2.50(1.94, 3.22)^a^	2.10(1.62, 2.72)^a^
Clear-cell	1.09(0.81, 1.48)	1.16(0.77, 1.76)	1.59(1.31, 1.94)^a^	1.55(1.20, 2.00)^a^
Endometrioid	0.56(0.40, 0.78)^a^	0.68(0.47, 0.98)^b^	0.50(0.39, 0.64)^a^	0.95(0.72, 1.26)
Others	0.76(0.56, 1.04)	1.21(0.93, 1.58)	1.09(0.99, 1.21)	1.13(1.03, 1.24)^b^
Surgery				
Yes	Ref.	Ref.	Ref.	Ref.
No	0.17(0.11, 0.25)^a^	0.22(0.15, 0.34)^a^	0.34(0.30, 0.40)^a^	0.40(0.36, 0.45)^a^

a: *p***<**0.01; b: *p* <0.05.

**Table 5:** Subgroup analyses stratified by TNM stage for all EOC patients (EOCSS)

	Stage I	Stage II	Stage III	Stage IV
Marital status				
Married	Ref.	Ref.	Ref.	Ref.
Divorced/separated	1.35(0.89, 2.05)	1.01(0.68, 1.51)	1.16(1.02, 1.31)^b^	1.12(0.99, 1.27)
Widowed	1.82(1.24, 2.68)^a^	1.38(0.97, 1.95)	1.13(1.00, 1.27)^b^	1.08(0.96, 1.21)
Never married	1.09(0.78, 1.50)	1.60(1.17, 2.19)^a^	1.02(0.91, 1.15)	1.13(1.00, 1.27)^b^
Age				
<60	Ref.	Ref.	Ref.	Ref.
60-69	0.72(0.50, 1.05)	0.98(0.70, 1.36)	1.18(1.06, 1.30)^a^	1.10(0.99, 1.23)
70-79	1.67(1.15, 2.43)^a^	1.88(1.33, 2.66)^a^	1.48(1.32, 1.66)^a^	1.14(1.01, 1.29)^b^
≥80	2.53(1.61, 3.95)^a^	2.31(1.56, 3.43)^a^	1.97(1.70, 2.28)^a^	1.51(1.31, 1.74)^a^
Race				
White	Ref.	Ref.	Ref.	Ref.
Black	1.03(0.65, 1.63)	1.44(0.96, 2.16)	1.17(1.00, 1.35)^b^	1.02(0.89, 1.16)
Others	0.76(0.51, 1.14)	0.75(0.48, 1.18)	0.95(0.82, 1.09)	0.98(0.84, 1.13)
Diagnosis year				
2004-2008	Ref.	Ref.	Ref.	Ref.
2009-2012	0.79(0.60, 1.04)	0.99(0.76, 1.28)	0.93(0.86, 1.01)	0.95(0.88, 1.04)
Grade				
I	Ref.	Ref.	Ref.	Ref.
II	1.58(0.96, 2.60)	1.21(0.61, 2.38)	1.46(1.02, 2.07)^b^	1.42(0.88, 2.28)
III	2.31(1.42, 3.76)^a^	1.87(0.97, 3.62)	1.91(1.37, 2.65)^a^	1.33(0.85, 2.07)
IV	2.09(1.18, 3.69)^b^	1.47(0.73, 2.96)	1.78(1.27, 2.49)^a^	1.28(0.81, 2.01)
Unknown	1.86(1.13, 3.07)^b^	1.76(0.89, 3.50)	1.88(1.34, 2.63)^a^	1.22(0.78, 1.91)
Histological type				
Serous	Ref.	Ref.	Ref.	Ref.
Mucinous	0.92(0.60, 1.41)	1.30(0.71, 2.37)	2.55(1.96, 3.32)^a^	1.99(1.52, 2.61)^a^
Clear-cell	1.07(0.75, 1.51)	1.36(0.88, 2.12)	1.60(1.31, 1.97)^a^	1.55(1.19, 2.01)^a^
Endometrioid	0.42(0.27, 0.65)^a^	0.64(0.41, 0.98)^b^	0.49(0.38, 0.63)^a^	0.88(0.65, 1.19)
Others	0.72(0.50, 1.04)	1.33(0.99, 1.77)	1.11(1.00, 1.23)	1.12(1.01, 1.23)^b^
Surgery				
Yes	Ref.	Ref.	Ref.	Ref.
No	0.11(0.07, 0.17)^a^	0.16(0.11, 0.26)^a^	0.34(0.29, 0.39)^a^	0.39(0.35, 0.44)^a^

a: *p* <0.01; b: *p* <0.05.

After adjusting for other covariates in Cox regression, patients with different marital status at stage IV showed similar risk of all-cause mortality; widowed patients had greater all-cause mortality risk than married ones at stage I, II, and III; divorced/separated showed prognostic disadvantage at stage I and III, while never married patients only at stage II. For EOC-specific survival, never married patients had poorer prognosis at stage II and IV; widowed patients showed disadvantage at stage I and III; while divorced/separated had greater cancer-specific mortality risk at stage III.

### TNM stage-stratified analyses of patients who underwent surgical procedures

**Figure 5:**
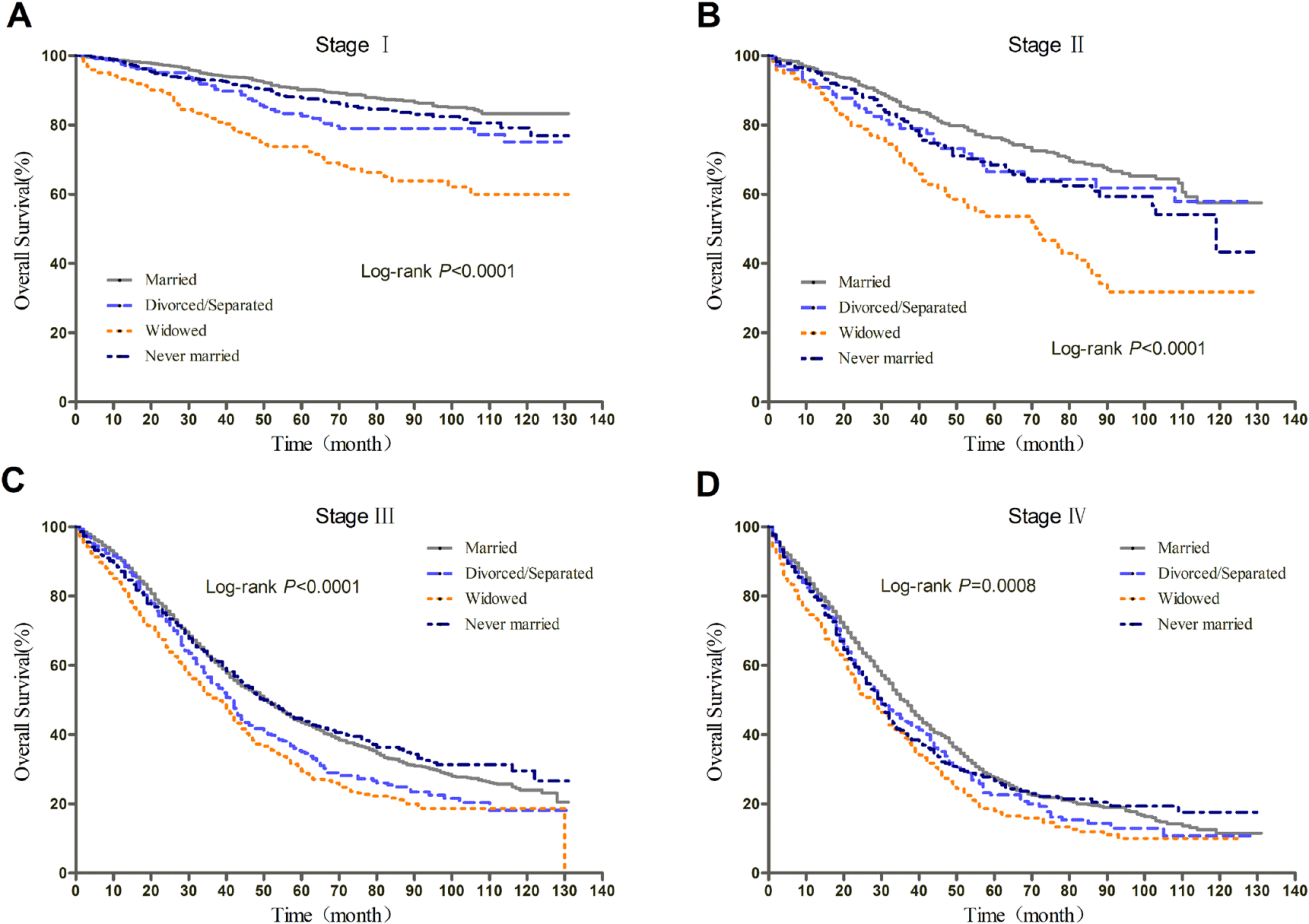
Kaplan-Meier curves of the effect of marital status on OS for surgical patients stratified by stage

**Figure 6:**
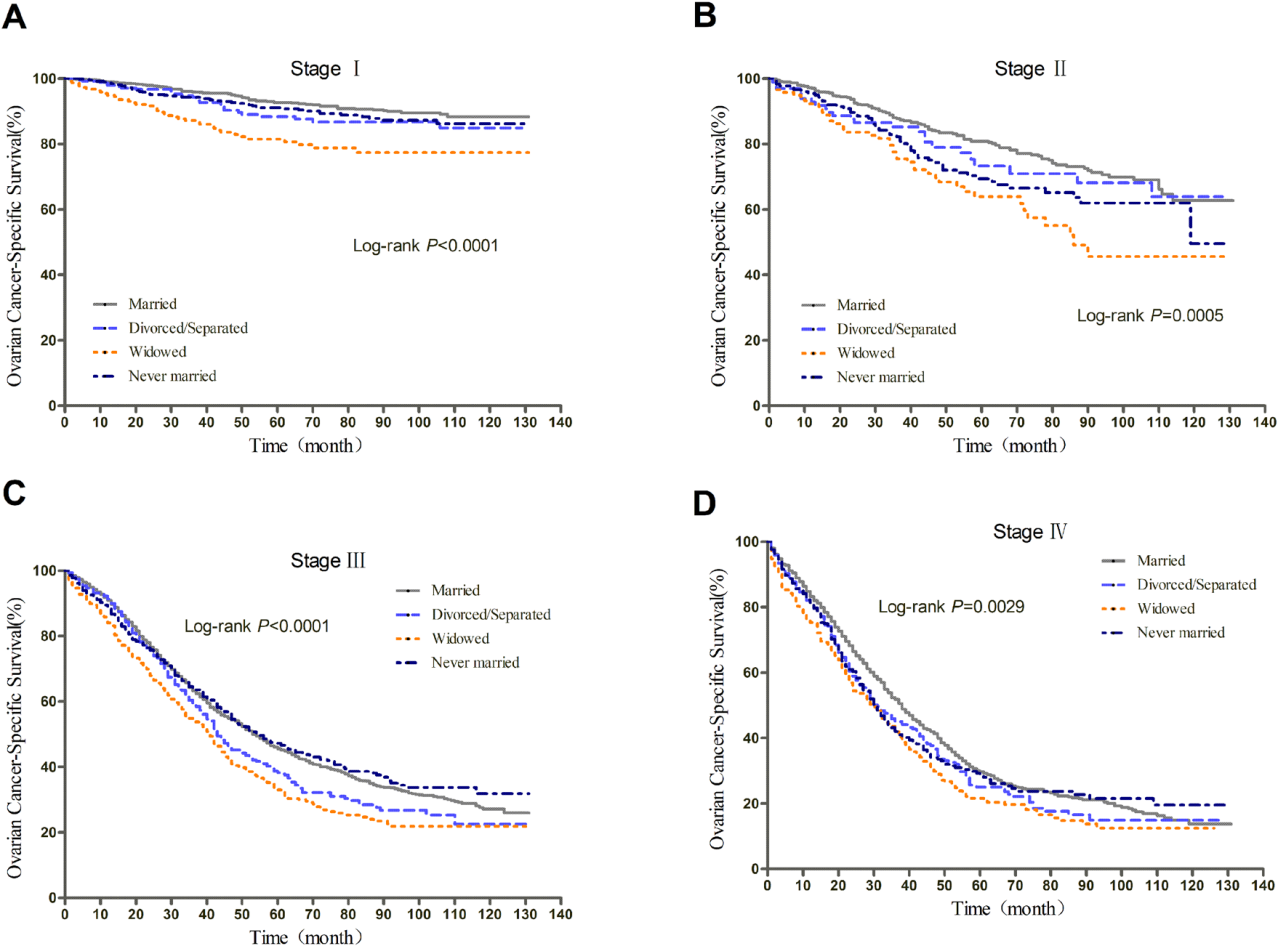
Kaplan-Meier curves of the effect of marital status on EOCSS for surgical patients stratified by stage

We also explored the effect of marital status on OS and EOCSS, stratified by TNM, in patients who received any type of surgery. The survival curves of the OS and EOCSS of different marital status were shown in Figure 5 and Figure 6, respectively. The results were summarized in Table 6 for OS and Table 7 for EOCSS.

**Table 6:** Subgroup analyses stratified by TNM stage for surgical patients (OS)

	Stage I	Stage II	Stage III	Stage IV
Marital status				
Married	Ref.	Ref.	Ref.	Ref.
Divorced/separated	1.56(1.10, 2.20)^b^	1.20(0.81, 1.78)	1.22(1.07, 1.39)^a^	1.15(0.99, 1.35)
Widowed	1.49(1.06, 2.10)^b^	1.30(0.92, 1.83)	1.07(0.94, 1.23)	1.18(1.00, 1.38)^b^
Never married	1.45(1.10, 1.92)^a^	1.49(1.09, 2.03)^b^	1.03(0.91, 1.16)	1.15(0.99, 1.33)
Age				
<60	Ref.	Ref.	Ref.	Ref.
60-69	1.10(0.81, 1.50)	1.10(0.80, 1.51)	1.24(1.12, 1.37)^a^	1.14(1.01, 1.30)^b^
70-79	2.49(1.80, 3.44)^a^	2.06(1.47, 2.88)^a^	1.66(1.48, 1.87)^a^	1.20(1.03, 1.39)^b^
≥80	5.13(3.52, 7.47)^a^	4.43(3.05, 6.43)^a^	2.15(1.81, 2.55)^a^	1.57(1.26, 1.94)^a^
Race				
White	Ref.	Ref.	Ref.	Ref.
Black	1.32(0.89, 1.96)	2.13(1.39, 3.25)^a^	1.21(1.03, 1.43)^b^	1.11(0.93, 1.33)
Others	0.80(0.56, 1.14)	0.82(0.54, 1.26)	1.02(0.88, 1.19)	1.01(0.85, 1.21)
Diagnosis year				
2004-2008	Ref.	Ref.	Ref.	Ref.
2009-2012	0.84(0.65, 1.08)	1.19(0.92, 1.56)	0.92(0.84, 1.00)	0.94(0.84, 1.05)
Grade				
I	Ref.	Ref.	Ref.	Ref.
II	1.41(0.96, 2.06)	1.30(0.73, 2.32)	1.46(1.04, 2.05)^b^	1.28(0.79, 2.09)
III	1.70(1.15, 2.51)^a^	1.65(0.95, 2.89)	1.83(1.33, 2.51)^a^	1.23(0.78, 1.95)
IV	1.49(0.93, 2.39)	1.25(0.68, 2.28)	1.69(1.22, 2.34)^a^	1.23(0.77, 1.97)
Unknown	1.56(1.03, 2.36) ^b^	1.78(0.99, 3.20)	1.74(1.25, 2.42)^a^	1.15(0.72, 1.84)
Histological type				
Serous	Ref.	Ref.	Ref.	Ref.
Mucinous	0.95(0.66, 1.37)	1.48(0.86, 2.56)	2.55(1.94, 3.34)^a^	1.97(1.42, 2.74)^a^
Clear-cell	1.14(0.83, 1.55)	1.18(0.77, 1.79)	1.54(1.25, 1.89)^a^	1.38(1.05, 1.82)^b^
Endometrioid	0.57(0.40, 0.81)^a^	0.69(0.47, 1.00)	0.49(0.38, 0.63)^a^	0.87(0.65, 1.17)
Others	0.77(0.54, 1.08)	1.17(0.88, 1.57)	1.05(0.93, 1.17)	0.96(0.84, 1.09)

a<0.01; b<0.05.

**Table 7:** Subgroup analyses stratified by TNM stage for surgical patients (EOCSS)

	Stage I	Stage II	Stage III	Stage IV
Marital status				
Married	Ref.	Ref.	Ref.	Ref.
Divorced/separated	1.42(0.92, 2.19)	1.10(0.70, 1.71)	1.16(1.02, 1.33)^b^	1.15(0.98, 1.35)
Widowed	1.81(1.18, 2.78)^a^	1.35(0.91, 2.00)	1.07(0.94, 1.24)	1.19(1.01, 1.41)^b^
Never married	1.38(0.99, 1.92)	1.61(1.16, 2.25)^a^	1.02(0.90, 1.15)	1.16(0.99, 1.35)
Age				
<60	Ref.	Ref.	Ref.	Ref.
60-69	0.76(0.52, 1.12)	1.04(0.74, 1.47)	1.21(1.09, 1.34)^a^	1.13(0.99, 1.29)
70-79	1.69(1.13, 2.53)^b^	1.86(1.29, 2.70)^a^	1.55(1.37, 1.75)^a^	1.13(0.97, 1.32)
≥80	2.25(1.33, 3.82)^a^	2.66(1.69, 4.18)^a^	1.99(1.67, 2.38)^a^	1.44(1.15, 1.81)^a^
Race				
White	Ref.	Ref.	Ref.	Ref.
Black	1.15(0.69, 1.92)	2.12(1.35, 3.34)^a^	1.17(0.99, 1.40)	1.11(0.92, 1.34)
Others	0.76(0.50, 1.16)	0.70(0.43, 1.15)	0.98(0.84, 1.15)	0.92(0.76, 1.11)
Diagnosis year				
2004-2008	Ref.	Ref.	Ref.	Ref.
2009-2012	0.76(0.57, 1.03)	1.14(0.85, 1.53)	0.92(0.84, 1.01)	0.94(0.84, 1.05)
Grade				
I	Ref.	Ref.	Ref.	Ref.
II	1.52(0.92, 2.52)	1.31(0.66, 2.61)	1.41(0.99, 2.00)	1.33(0.80, 2.21)
III	2.29(1.39, 3.77)^a^	1.93(0.99, 3.76)	1.83(1.31, 2.55)^a^	1.25(0.77, 2.02)
IV	2.00(1.12, 3.57)^b^	1.51(0.74, 3.07)	1.70(1.22, 2.39)^a^	1.27(0.78, 2.07)
Unknown	1.70(1.00, 2.88)	1.92(0.96, 3.83)	1.77(1.26, 2.50)^a^	1.16(0.71, 1.90)
Histological type				
Serous	Ref.	Ref.	Ref.	Ref.
Mucinous	0.93(0.60, 1.45)	1.79(0.99, 3.23)	2.57(1.94, 3.40)^a^	1.86(1.31, 2.63)^a^
Clear-cell	1.10(0.77, 1.58)	1.41(0.90, 2.20)	1.54(1.25, 1.91)^a^	1.41(1.06, 1.88)^b^
Endometrioid	0.43(0.27, 0.67)^a^	0.64(0.42, 1.00)^b^	0.48(0.37, 0.62)^a^	0.81(0.59, 1.11)
Others	0.68(0.45, 1.03)	1.29(0.93, 1.77)	1.07(0.95, 1.20)	0.95(0.82, 1.09)

a: *p* <0.01; b: *p* <0.05.

For patients at Stage I, all the three unmarried groups had poorer prognosis when compared with the married group; additionally, never married patients at stage II, divorced/separated patients at stage III, and widowed patients at stage IV were identified to have greater all-cause mortality risk than married patients. In the context of EOCSS, widowed patients at stage I and IV, never married patients at stage II, and divorced/separated patients at stage III were discovered in the multivariate Cox regression to have survival disadvantage over married patients.

## DISCUSSION

In this study, we found that unmarried patients, including divorced/separated, widowed and never married, were at significantly greater risk of mortality after diagnosis of EOC. After adjusting for demographic factors, clinical characteristics, and treatment, complete marriage was still associated with a reduction of the risk of death. When we analyzed the effect of marital status on OS and EOCSS stratified by stage, in overall patients and patients having surgery, the effect differed according to different conditions. Moreover, the results showed that widowed patients had the highest proportion of being diagnosed at advanced stage and the lowest proportion of receiving surgical treatment.

Though most of the findings of this study are consistent with the findings of previous observational studies conducted on other types of cancer [8, 12, 17, 18], that unmarried patients had survival disadvantage compared with married patients, the significance of this study is that we analyzed the effect of marital status on the OS and EOCSS according to different stages and repeated the analyses for patients who underwent surgery, which had never been investigated for ovarian cancer before.

Moreover, contrary to the statement that married patients are more likely to be diagnosed at early stage [7, 9], we found that the never married patients had the highest proportion of being diagnosed at stage I (30.31%) and the second highest proportion of receiving surgical treatment. What is astounding is that the never married patients had the highest 5-year overall survival and cancer-specific survival, slightly higher than the married ones. The phenomenon may be explained by several reasons. The first is that the never married group of this study included the single, unmarried or domestic partner (same sex or opposite sex or unregistered), which decreased the proportion of truly unmarried patients in that group and made the results underestimate the effect of being truly unmarried. Besides, the ones that actually had a partner in this group may enjoy a more satisfied relationship even though they did not have a registered marriage. Second, since that most of the never married group were younger than 60 years (67.41%), an age of sufficient vigilance of any sign of disease, it was reasonable that they visited doctors and detected the tumor earlier. Third, the never married patients had very less possibility of having given birth to a child, thus may pay more attention to their reproductive system. However, after adjusting for other confounders in the multivariate Cox analyses, the never married patients did not show as much advantage as the married ones. They went through nearly the same amount of risk as the widowed patients for all-cause mortality (HR: 1.11 for the never married vs 1.12 for the widowed), and cancer-specific mortality (HR: 1.11 for the never married vs 1.12 for the widowed). It is possible that although some of the patients in the never married group were actually accompanied, the truly single ones may suffer more than the widowed, since widowed patients can obtain the physical and psychological support from their children. Therefore, the similar hazard ratio may be a compromise of the two kinds of situation in the never married group.

There are many existing explanations of the benefit of marriage for patients and they are also suitable for cancer patients. Generally, marriage, which is a main source of social support, can build up the patients’ confidence to conquer the disease, increase the patients’ adherence to the treatment plans and prescriptions of doctors [19], which can directly lead to better prognosis [20–22]. It can also lower the level of depression and anxiety [23, 24]. Female patients may suffer more from the loss of support than male patients. Therefore, policy makers and doctors should make efforts to improve the social support from every aspect for the EOC patients who are in unmarried status.

The SEER database provides us the opportunity to perform large, population-based studies. However, there are several vital limitations that should be addressed and the results of the study should be interpreted with caution. Firstly, this is an observational study and the results cannot be used to conduct causal inferences. We cannot state that the unmarried status lead to the poorer prognosis of patients, but we can conclude that the marital status is associated with, or a predictor of, the EOC prognosis. Further analyses should explore the contribution magnitude, in both direct and indirect way, of the role of marital status to the survival of EOC after diagnosis. Secondly, the limited number of accessible variables may lead to the overestimation of the effect of marital status on the cancer survival. Many other aspects can also play important roles in cancer prognosis such as comorbidity, chemotherapy, and radiotherapy. Thirdly, the follow-up period for some of the patients was so long that the marital status probably changed during the study. This is especially possible for elder patients, who were married at diagnosis but became widowed afterwards. Besides, the quality of marriage was unknown. Therefore, the classification of marriage was nominal and relatively rough.

Despite these limitations, our study indicates that unmarried EOC patients including divorced/separated, widowed, and never married, are at greater risk of overall mortality and EOC-specific mortality. When caring for the unmarried cancer patients, medical staff should be well aware of the potentially poorer prognosis in this population and health care systems should make efforts to provide social support for this population to minimize the risk of death caused by marital status.

## MATERIALS AND METHODS

### Data source

In this study, we utilized the data obtained from SEER program, which consists of 18 registries covering approximately 28 percent of the US people and routinely collecting information of cancer patients including demographics, primary tumor site, cancer stage, treatment and the follow up information of survival. The database is an authoritative source of information on the incidence and survival of cancer in the United States and has been used by many studies to search prognostic factors associated with various cancers [25–28].

### Inclusion criteria

Patients with ICD-O-3 (International Classification of Diseases for Oncology, 3^rd^ edition) site code C56.9 from 2004 to 2012 were extracted from the SEER database. The inclusion criteria were as follows: (a) ICD-O-3 morphology code indicated epithelial ovarian cancer;(b) age at diagnosis was older than 18 years; (c) marital status was known; (d) diagnosed with EOC only or multiple primary cancers but EOC was the first; (e) known survival time and survival time was greater than 0 month; (f) known cause of death;(g) definite AJCC (American Joint Committee on Cancer) stage group, 6^th^ ed TNM stage (h) known surgery information.

### Study variables and outcomes

Study variables in this study included age at diagnosis, gender, year of diagnosis, race, histologic type, tumor grade, AJCC TNM stage, and surgical information. The patients were divided into four groups according to age (<60, 60-69,70-79, and ≥80). Marital status was classified as married, divorced or separated, widowed, and never married (including single, unmarried or domestic partner). Race was grouped by white, black, and others (including American Indian/Alaska native, Asian/Pacific Islanders, etc.). We divided the year of diagnosis into 2004-2008 and 2009-2012 to adjust for the survival difference caused by the advances of diagnostic techniques and therapeutic methods with the passage of time. Histologic type was classified as serous, mucinous, clear-cell, endometroid, and other epithelial types. Tumor grade I-IV represented well differentiated, moderately differentiated, poorly differentiated, and undifferentiated, respectively. Surgery was categorized as yes (received any type of surgery) and no (did not receive surgery).

The primary outcomes were overall survival (OS) and epithelial ovarian cancer-specific survival (EOCSS). OS was calculated as the number of month from diagnosis to death due to any cause. EOCSS was calculated as the number of month from diagnosis to death due to EOC. Patients who died from other causes or were still alive at the end of the study period were defined as censored.

### Statistical analyses

The baseline characteristics of patients with different marital status were summarized and compared using chi-square test. Kaplan-Meier log-rank test was adopted to compare the difference of OS and EOCSS between subgroups of each variable. Multivariate Cox analysis was conducted to compare the OS and EOCSS of patients with different marital status after adjusting for various covariates. To explore how marital status affected patients diagnosed at the same stage, we further conducted subgroup analyses, stratified by different AJCC TNM stage, in all patients and patients who underwent surgical procedures. All P values were two-sided, and the values less than 0.05 were considered statistically significant. All analyses were performed using SAS version 9.4 (SAS Institute Inc., Cary, NC, USA). All figures were created with Graphpad Prism 5.0.

## Abbreviations

EOC: epithelial ovarian cancer
OS: overall survival
EOCSS: epithelial ovarian cancer-specific survival
AJCC: American Joint Committee on Cancer
